# Changes in white matter microstructure in the developing brain—A longitudinal diffusion tensor imaging study of children from 4 to 11 years of age

**DOI:** 10.1016/j.neuroimage.2015.09.017

**Published:** 2016-01-01

**Authors:** Stine K. Krogsrud, Anders M. Fjell, Christian K. Tamnes, Håkon Grydeland, Lia Mork, Paulina Due-Tønnessen, Atle Bjørnerud, Cassandra Sampaio-Baptista, Jesper Andersson, Heidi Johansen-Berg, Kristine B. Walhovd

**Affiliations:** a Research Group for Lifespan Changes in Brain and Cognition, Department of Psychology, University of Oslo, Norway; b Department of Physical Medicine and Rehabilitation, Unit of Neuropsychology, Oslo University Hospital, Norway; c Department of Radiology, Rikshospitalet, Oslo University Hospital, Norway; d The Interventional Centre, Rikshospitalet, Oslo University Hospital, Norway; e The Oxford Centre for Functional MRI of the Brain (FMRIB), Nuffield Department of Clinical Neurosciences, University of Oxford, John Radcliffe Hospital, Oxford, UK

**Keywords:** WM, White matter, DTI, Diffusion tensor imaging, FA, fractional anisotropy, MD, Mean diffusivity, RD, Radial diffusivity, AD, Axial diffusivity, LCBC, Research Group for Lifespan Changes in Brain and Cognition, MoBa, Norwegian Mother and Child Cohort Study, tp1, Time point one, tp2, Time point two, MRI, Magnetic resonance imaging, CNS, Central nervous system, TR, Repetition time, TE, Echo time, GRAPPA, GeneRalized Autocalibrating Partially Parallel Acquisition, FMRIB, Oxford Centre for Functional Magnetic Resonance Imaging of the Brain, TBSS, Tract-Based Spatial Statistics, GLM, General linear model, SDR, Studentized Deleted Residuals, GAMM, General additive mixed model, JHU, Johns Hopkins University, ATR, Anterior thalamic radiation, CCG, Cingulum-cingulate gyrus, CHG, Cingulum-hippocampus gyrus, CC, corpus callosum, IFOF, Inferior fronto-occipital fasciculus, ILF, Inferior longitudinal fasciculus, SLF, Superior fronto-occipital fasciculus, SFOF, Superior fronto-occipital fasciculus, UF, Uncinate fasciculus, Development, White matter maturation, MRI, DTI, Longitudinal, Children

## Abstract

The purpose of the present study was to detail the childhood developmental course of different white matter (WM) characteristics. In a longitudinal diffusion tensor imaging (DTI) study of 159 healthy children between 4 and 11 years scanned twice, we used tract-based spatial statistics as well as delineation of 15 major WM tracts to characterize the regional pattern of change in fractional anisotropy (FA), mean (MD), radial (RD) and axial diffusivity (AD). We tested whether there were decelerations of change with increasing age globally and tract-wise, and also illustrated change along medial-to-lateral, posterior-to-anterior and inferior-to-superior gradients. We found a significant linear increase in global FA, and decrease in MD and RD over time. For mean AD, a weak decrease was observed. The developmental changes in specific WM tracts showed regional differences. Eight WM tracts showed non-linear development patterns for one or several DTI metrics, with a deceleration in change with age. Sex did not affect change in any DTI metric. Overall, greater rate of change was found in the left hemisphere. Spatially, there was a posterior-to-anterior gradient of change with greater change in frontal regions for all metrics. The current study provides a comprehensive characterization of the regional patters of change in WM microstructure across pre-adolescence childhood.

## Highlights

• We explore longitudinal change in white matter (WM) microstructure in children.
• Using TBSS to delineate 15 WM tracts and several gradients in the brain
• Global WM change show linear developmental patterns from age 4 to 11 years.
• Greater WM change in the anterior compared to the posterior brain region.
• The developmental patterns for WM tracts show regional differences.

## Introduction

White matter (WM) makes up about half the human brain, and being the prime conductor of neural signaling has a pivotal role in the development of human behavior. WM maturation is known to be prolonged, yet the specific developmental course of different WM characteristics remains elusive (Giedd et al., 1999, Lenroot et al., 2007, Lebel and Beaulieu, 2011). Specifically, how the microstructural connectivity changes in the preschool and early school years proceed along major WM tracts, and how they can be described along major spatial gradients in the brain, i.e. posterior-to-anterior, medial-to-lateral and inferior-to-superior, have not been thoroughly characterized longitudinally. This will be addressed in the present study.

WM microstructure has been found to change rapidly in infancy (Mukherjee et al., 2001, Hermoye et al., 2006, Dubois et al., 2008, Geng et al., 2012). Concerning late childhood and adolescence, cross-sectional developmental studies have documented age-related fractional anisotropy (FA) increases and overall diffusivity decreases with age in most WM regions (Lebel et al., 2008, Schmithorst and Yuan, 2010, Tamnes et al., 2010, Peters et al., 2012). These age-related differences are thought to relate to neurobiological processes including increased relative axon caliber and myelin content, as well as changes in fiber packing density (Beaulieu, 2002, Paus, 2010, Simmonds et al., 2014). A few longitudinal studies are now also confirming widespread WM FA increases, and mean (MD) and radial (RD) diffusivity decreases through late childhood and adolescence, but the results for axial diffusivity (AD) are less consistent (Bava et al., 2010, Giorgio et al., 2010, Lebel and Beaulieu, 2011, Brouwer et al., 2012). Development of diffusion directionality and magnitude in WM, possibly related to an underlying increase in the diameter and myelination of axons, among other factors, may play a role in cognitive development during childhood and adolescence (Johansen-Berg, 2010, Vestergaard et al., 2011, Peters et al., 2014).

While white matter changes with age may be both global as well as tract-specific, a consideration of regional age changes regardless of the often long-ranging specific tracts may be of interest. There is evidence to suggest possibly broad regional differences in brain maturation. For instance, for cortical gray matter, a posterior–anterior sequence of maturation has repeatedly been identified (Gogtay et al., 2004, Tzarouchi et al., 2009, Tamnes et al., 2010), and WM development varies regionally in the brain (Lebel et al., 2012). White matter maturation, including myelination, starts prenatally and appears to progress in an orderly manner during infancy from posterior-to-anterior, inferior-to-superior, and central-to-peripheral regions (Barkovich et al., 1988, Bendersky et al., 2006, de Graaf-Peters and Hadders-Algra, 2006). Later systematic regional age-related WM differences have been investigated *in vivo* using DTI (Tamnes et al., 2010, Westlye et al., 2010b, Colby et al., 2011, Lebel and Beaulieu, 2011, Lebel et al., 2012). In a cross sectional study, Colby et al. (2011) demonstrated gradients in the developmental timing of white matter maturation, as measured by FA along inferior-to-superior and posterior-to-anterior directions from 5 to 28 years. Westlye et al. (2010a) showed that intra-cortical T1 signal intensity followed a posterior-to-anterior gradient from childhood to adulthood. In adults, age-related changes have been found to increase gradually by posterior–anterior and inferior–superior gradients (Sexton et al., 2014). Prefrontal WM has shown reduced FA in aging (Salat et al., 2005), and an anterior to posterior gradient of degeneration has been suggested with support from several studies (Pfefferbaum et al., 2000, Head et al., 2004, Bennett et al., 2010). However, to our knowledge, no longitudinal studies have systematically described change-patterns along the primary gradients in the developing brain as a supplement to the tract specific changes.

Here we address longitudinal development of structural brain connectivity in 159 participants from 4 to 11 years. We hypothesized 1) developmental increases in FA along with decreases in MD, RD and to a lesser extent in AD throughout the FA skeleton (Bava et al., 2010, Tamnes et al., 2010, Lebel and Beaulieu, 2011, Simmonds et al., 2014), 2) deceleration of change with increasing age (Lebel et al., 2008, Lebel and Beaulieu, 2011), 3) some variations in change rates across different WM tracts due to the rapid development of specific tracts in early postnatal life (Hermoye et al., 2006, Uda et al., 2015), and 4) increased change may also be observed along the posterior-to-anterior (Tamnes et al., 2010, Westlye et al., 2010a, Colby et al., 2011), inferior-to-superior (Sexton et al., 2014) and medial-to-lateral gradients (Hermoye et al., 2006).

## Methods

### Participants

All participants were recruited from the Norwegian Mother and Child Cohort Study (MoBa) (Magnus et al., 2006) undertaken by the Norwegian Institute of Public Health to the project, run by the Research Group for Lifespan Changes in Brain and Cognition (LCBC) at the Department of Psychology, University of Oslo, Norway. The project was approved by the Regional Committee for Medical and Health Research Ethics. Written informed consent was obtained from the parent/guardian for all participants and oral assent was given by participants at both time points.

Two hundred and ninety-six children met the inclusion criteria (see below) and underwent DTI scanning at time point one (tp1). Of these, 173 completed DTI scans at both time points, yielding a total of 123 dropouts from tp1 to time point 2 (tp2). The main reasons for drop out were the parent's busy schedule (n = 45). Additionally, 21 children did not want to participate, 11 of the families had moved, 10 parents did not want their child to undergo magnetic resonance imaging (MRI) a second time, and 35 were not able to participate due to other circumstances. One child did not participate due to undisclosed health reasons at tp2. Of the 173 that had DTI scans at both time points, 14 participants (mean age = 5.5, SD = 1.0, 8 females) were excluded based on motion artifacts (see Motion parameters section): 10 of whom based on motion at tp1 and 4 based on motion at tp2.

A parent of each participant completed a structured interview to ascertain participant eligibility at both time points. Included participants were required to be fluent Norwegian speakers and have normal or corrected-to normal vision and normal hearing. Exclusion criteria were history of injury or disease known to affect central nervous system (CNS) function, including neurological or psychiatric illness, serious head trauma such as been unconscious, being under psychiatric treatment, use of psychoactive drugs known to affect CNS functioning, low birth weight (< 2500 g), and MRI contraindications. Participants recruited for the study were not excluded based on handedness (left-handed participants N = 11, mean age = 6.5, SD = 1.2). All participants' scans were also examined by a neuroradiologist and required to be deemed free of significant injuries or pathological conditions at both time points. One participant did not meet this inclusion criterion at tp1.

Briefly, 159 participants (90 females) had longitudinal data and were included in this study. At tp1 the age range was from 4.2 to 9.3 (Mean = 6.2, Median = 6.0, SD = 1.1), and at tp2 the age ranged from 5.8 to 11.0 (Mean = 7.8, Median = 7.6, SD = 1.1). Mean interval between scans was 584 days (SD = 52), ranging from 456 to 819 days. Interval between scans was not significantly correlated with age at tp1 (r = .12, p = .131), but was at tp2 (r = .24, p = .002), and was not different for females and males (t = − 1.88, p = .063). All participants > 6.5 years of age tested within normal range on estimated IQ (Wechsler, 1999) at tp1 (77–140, M = 109.9, SD = 11.7) and tp2 (77–141, M = 108.8, SD = 12.1), and participants ≤ 6.5 years of age tested within normal range on scaled general cognitive measures (Wechsler, 2002) at tp1 (6–16, M = 11.6, SD = 1.9) and tp2 (11–15, M = 12.3, SD = 1.3). Participant characteristics for the final sample are provided in Table 1.

At tp1, all children underwent a practice session in a mock scanner to get familiarized with the procedures, the small space and the sounds of the MRI-scanner. They were also shown a video recorded at Oslo University Hospital with a child going through each step of the MRI session. This was also done at tp2 for the children that expressed concern related to the MRI session.

### MRI acquisition

All MRI data was collected using a 12-channel head coil on a 1.5 T Siemens Avanto scanner (Siemens Medical Solutions) at Rikshospitalet, Oslo University Hospital. The same scanner, head coil and sequences were used at both time-points, though with a software upgrades from B17 to B19 for most participants at tp2 (n = 136). DTI was performed with the following parameters: repetition time (TR) = 8200 ms; echo time (TE) = 81 ms; voxel size = 2.0 mm isotropic; number of slices = 64; FOV = 128; matrix size = 128 × 128 × 64; b value = 700 s/mm; number of diffusion weighted directions = 32; number of b0 images = 5 (the first 33 participants were scanned with b0 = 1); A GeneRalized Autocalibrating Partially Parallel Acquisition (GRAPPA) factor of 2 was used. Acquisition time was 5 min 30 s.

### Motion parameters

Data was corrected for eddy current-induced distortions and subject movement (Andersson et al., 2012, Sotiropoulos et al., 2013). In short, this procedure uses all diffusion weighted volumes to make a prediction (based on a Gaussian Process) what each volume “should look like” and then registers the observed volumes to that prediction using a rigid body model for the movements and assuming a first order eddy current-induce field. In some of these data sets there was signal drop-out. This is caused by a rotation (subject movement) coinciding exactly in time with the diffusion encoding and shows itself as multiplicative signal dropout across the entire slice that was affected by the movement. It can also be caused by pulsatile movement leading to a local rotation which will then manifest as a local dropout typically around the brain stem area. The eddy current correction method has been extended to also use signal dropouts by comparing the observed slice to the predicted and deciding if the difference is large enough to make it an outlier among all such differences (Andersson and Sotiropoulos, 2014). If a slice is determined to constitute an outlier it is removed and the prediction is recalculated without the offending slice and the new prediction is inserted as a replacement for the removed slice. Only scans deemed to have no or minimal movement artifacts were included in the analyses. Based on the eddy outlier report and manual checking, all volumes > 10 slices of signal dropout detected by the eddy correction method were deemed bad. For participants (n = 85) with 1–6 bad volumes, we excluded the bad volumes and re-corrected for eddy current-induced distortions and subject movement. This was especially done for participants with sudden motion in the scanner. Participants exceeding 6 bad volumes were excluded from the study (see Participants section).

### MRI analysis

Analyses were performed at the Neuroimaging Analysis Laboratory, LCBC, University of Oslo and at the Oxford Centre for Functional Magnetic Resonance Imaging of the Brain (FMRIB), University of Oxford. Analysis of DTI data was carried out using Tract-Based Spatial Statistics (TBSS; (Smith et al., 2006)), part of FSL (Smith et al., 2004). All DTI images were corrected for eddy-current-induced distortions and head motion by means of an affine registration to the reference (b0) volume (see Motion parameters) (Andersson and Sotiropoulos, 2014), and brain-extracted using BET (Smith, 2002). Then, the FA and eigenvalue maps were computed by fitting a tensor model to the diffusion data. All participants' FA data were then aligned into a common space using the nonlinear registration tool FNIRT in a process where every FA image was aligned to every other one (Andersson et al., 2007a, Andersson et al., 2007b), using a b-spline representation of the registration warp field (Rueckert et al., 1999). Next, the mean FA across participants and time points was created based on the FA image that had the smallest amount of average warping when used as a target. The target was affine-aligned into MNI152 standard space and this target-to-MNI152 affine transform was combined with each participant's nonlinear transform to the target. This single transform was then applied to each subject's FA image bringing each image into standard space in one transformation. The resulting standard space FA images were then averaged and thinned to create a mean FA skeleton which represents the centers of all tracts common to the group. The threshold for the mean FA skeleton was set at 0.25 to reduce the likelihood of partial voluming in the borders between tissue classes, yielding a mask of 152,284 WM voxels. Each participant's aligned FA data was then projected onto this skeleton by searching perpendicular from the skeleton for maximum FA values. We calculated maps of change between tp2 and tp1 (tp2 − tp1), and the resulting data was fed into voxelwise cross-subject statistics. The FA-derived nonlinear warps were applied to the MD, RD, and AD change maps and values were projected onto the skeleton from the same voxels as in the FA analysis (i.e. the voxel with highest FA perpendicular to each point on the skeleton). MD was defined as the mean of all three eigenvalues (λ1 + λ2 + λ3/3), RD as the mean of the second and third eigenvalues (λ2 + λ3/2), and AD as the principal diffusion eigenvalue (λ1).

Two probabilistic WM tractography atlases (the Johns Hopkins University (JHU) and JHU ICBM DTI White Matter Labels) (Mori et al., 2005) provided with FSL were used to extract diffusivity tract values with a probability threshold of 5%. The relatively liberal threshold was chosen to accommodate inter-subject variation in gross WM fiber architecture, and for the skeleton voxels to intersect the correct tract appropriately (Smith et al., 2006). DTI indices from the overlap between the FA skeleton and the following tracts were extracted: left and right anterior thalamic radiation (ATR), left and right cingulum-cingulate gyrus (CCG), left and right cingulum-hippocampus gyrus (CHG), corpus callosum (CC body, CC genu and CC splenium), left and right corticospinal tract (CST), forceps major, forceps minor, fornix, left and right inferior fronto-occipital fasciculus (IFOF), left and right inferior longitudinal fasciculus (ILF), left and right superior longitudinal fasciculus (SLF), left and right superior fronto-occipital fasciculus (SFOF), left and right uncinate fasciculus (UF). The fit of the atlas WM tracts were manually checked, and deemed satisfactory with only a minimal/negligible amount of non-tract of interest voxels included.

### Statistical analysis

Voxelwise statistics were performed on change maps using “randomize” with 5000 permutations to control the family-wise error rate (Nichols and Holmes, 2002). General linear model (GLM) analyses were run with age, sex, motion at both time points and interval as covariates to investigate change throughout the skeleton for FA, MD, RD and AD, respectively. We extracted the number of significant voxels and equivalent percentages (p < 0.05, after correction for multiple comparisons across space) in the FA skeleton for FA, MD, RD and AD when controlling for age, sex, motion at both time points and interval. Next, we ran the same GLM testing the effect of age on change with sex, motion at both time points and interval as covariates. All covariates were demeaned. Global hemisphere differences were also assessed, testing both left > right hemisphere and left < right hemisphere. Please see Supplementary Fig. 1 for results. In PASW Statistics 22 (SPSS, Chicago, IL), we ran partial correlations between global FA, MD, RD and AD at both time points and age, controlling for motion at each time point and sex. Additionally, to test the relationship between mean change for FA, MD, RD and AD (time point 2 − time point 1) and age, partial correlations were run with motion at both time points, sex and interval as covariates. Possible effects of the different predictors on change were also tested by running a GLM to test effects of age, sex, interval, motion at both time points and age x sex interactions on change for FA, MD, RD and AD, respectively.

To quantify possible outlier values, Studentized Deleted Residuals (SDR) from mean FA, MD, RD and AD values from both time points predicted by age were calculated. The partial correlations were recalculated after outlier analysis, excluding individuals exceeding SDR ± 3 to make sure that these participants did not unduly affect our results. Assessment of normality for global change in FA, MD, RD and AD was done by running the Shapiro–Wilk test, and all the variables were normally distributed. To illustrate change within individuals, spaghetti plots were created for mean FA, MD, RD and AD. As global fits, such as linear and quadratic models, may be affected by irrelevant factors, such as the sampled age range (Fjell et al., 2010), an assumption-free longitudinal nonparametric general additive mixed model (GAMM) for each measure as a function of age was fitted to accurately describe developmental trajectories across the studied age range. The model does not assume a linear relationship. Curve fitting was performed using functions freely available through the statistical environment R, version 3.0.1 (http://www.r-project.org/).

For illustration purposes, spaghetti plots of individual participant change in FA, MD, RD and AD in each specific tract were created and GAMM used to obtain a fit line combining longitudinal and cross-sectional information, without the inclusion of any covariates. Annual percentage change (APC) for global FA, MD, RD, AD and all WM tracts were calculated. To compare differences between global APC and APC in WM tracts, paired t-tests for all DTI metrics were performed for each tract separately. To test effects of sex for change in WM tracts, the GLM was repeated, controlling for age, motion at both time points and interval on change, and was Bonferroni-corrected by a factor of 15 (reflecting the fifteen extracted WM tracts). Paired-samples t-tests were performed to compare change in all bilateral tracts (left hemisphere–right hemisphere) in FA, MD, RD and AD, and were Bonferroni-corrected by a factor of 9 (reflecting the nine bilateral WM tracts).

To illustrate how developmental change rates varied along medial-to-lateral, posterior-to-anterior or inferior-to-superior gradients, mean change (tp2 − tp1) for FA, MD, RD and AD were extracted across all skeleton voxels for each coronal, sagittal and axial slice, excluding the most distal slices with < 500 voxels. The aim was not to test whether WM microstructure change primarily along major WM tracts versus along major spatial gradients. We then plotted the z-transformed change values across x, y and z co-ordinates in MNI space using robust LOESS (rLOESS) fitting in Matlab (Mathworks, Inc.) with span of 30%. We tested whether change was significantly different along gradients by creating a set of ROIs. We tested the medial-to-lateral gradient by averaging the most distal 25 x-coordinates in both left and right hemisphere (lateral), and contrasted this ROI with the remaining x-coordinates (medial). The posterior ROI and anterior ROI was split at y = 90 and averaged, and the inferior ROI and superior ROI was split at z = 73 and averaged. Paired t-tests were run to test for differences in change between ROIs. In addition, mean change for each gradient was calculated and t-tests were run to test for differences between mean change and change along each gradient (please see Fig. 7).

## Results

### Pattern of change

**Fig. 1:**
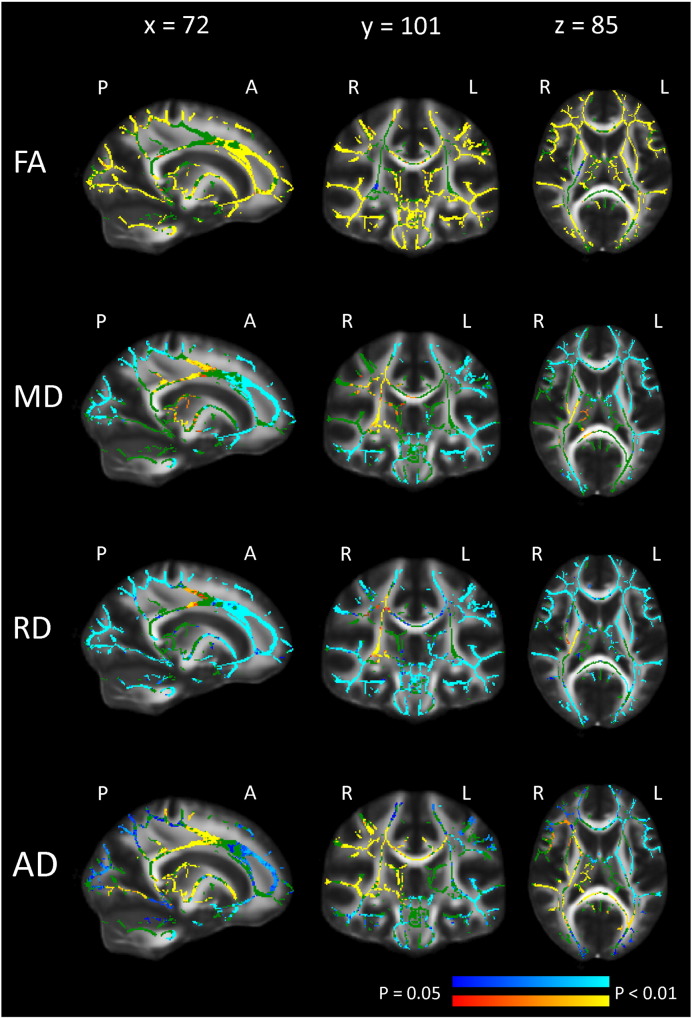
*Pattern of change for mean FA, MD, RD and AD.* Pattern of change controlling for motion at both time points, sex, age and interval. Regions with a significant (p < 0.05, correction for multiple comparisons across space) increase (yellow and red) and decrease (blue and light blue) in FA, MD, RD and AD are overlaid on the FA skeleton (green), displayed on the FMRIB FA template in MNI space. X, Y and Z are MNI coordinates. P = posterior, A = anterior, L = left hemisphere and R = right hemisphere.

Voxelwise analyses showed mainly significant increase in global FA, and decreases in global MD and RD between time points throughout most of the skeleton (see Fig. 1), controlling for age, sex, motion at both time points and interval. For global AD, the pattern was more complex. APC for global mean FA, MD, RD and AD were 1.80%, − 0.73%, − 1.49% and − 0.02%, respectively (Table 4), and the significant increase in FA and decrease in MD, RD and AD covered 73%, 51%, 67% and 18% of the skeleton, respectively (Table 2). Opposite effects, with a decrease in FA and increase in MD and RD, were observed to a small extent covering 0.1%, 5% and 2% of the skeleton, respectively. For AD, however, the numbers of voxels showing decrease vs. increase were almost equivalent (17% vs. 18%). Increase for FA was especially evident in association tracts (SLF, ILF, IFOF and UF), while some callosal fibers (CC splenium and parts of the forceps major) only showed significant increase in parts of the tracts. Also, a decrease in FA was found in small parts of the right CST. For MD, bilateral decrease was found for CHG, UF and forceps minor, while decrease in the left hemisphere and no change or increase was found in the right hemisphere for some WM tracts (CST, SLF and IFOF). The change pattern for RD was overall similar to that of MD, although with less hemisphere differences. AD showed strong lateralization patterns with mainly increases in the right hemisphere and decrease in the left hemisphere.

### Age-related patterns of change

There was no effect of age on change in any DTI metric in the skeleton, controlling for sex, motion at both time points and interval (see table 3). In cross-sectional analyses, a significant positive cross-sectional correlation with age was found for mean FA at tp1 (r = .34, p < .001) and tp2 (r = .30, p < .001), and significant negative correlations for mean MD at tp1 (r = − .27, p < .001) and tp2 (r = − .24, p = .003), mean RD at tp1 (r = − .31, p < .001) and tp2 (r = − .27, p ≤ .001), controlling for motion at each time point and sex. There were no significant correlations with age for mean AD at tp1 (r = − .13, p = .132) or tp2 (r = − .13, p = 104) controlling for motion at each time point and sex.

Results from the GLM showed no significant (p < 0.05) effect of age, sex, or motion at either time point, or age x sex interactions (ranging from p = .310 to p < .999) for change in FA, MD and RD. For MD, there was a trend toward an association between change and motion tp1 (F = 3.52, p ≤ .062) where less decrease in MD was associated with more motion, and motion at tp2 (F = 2.92, p ≤ .090) where more decrease in MD was associated with more motion, and for AD, there was an effect of motion at tp1 (F = 11.51, p ≤  .001) where less decrease in AD was associated with more motion, and motion at tp2 (F = 10.72, p ≤ .001) where more decrease in AD was associated with more motion.

Six participants had SDR values exceeding ± 3 (SDR ranged from − 4.07 to 4.44) on mean FA, MD, RD or AD values. Outlier analyses showed that exclusion of these 6 participants did not affect the results much (partial correlations with age, controlling for motion at both time points, sex and interval tp1/tp2 for; FA: r = .37; p = .001/r = .29; p = .001, MD: r = − .28; p = .001/r = − .21; p = .009, RD: r = − .32; p = .001/r = − .25; p = .002, AD: r = − .12; p = .134/r = − .11; p = .177).

**Fig. 2:**
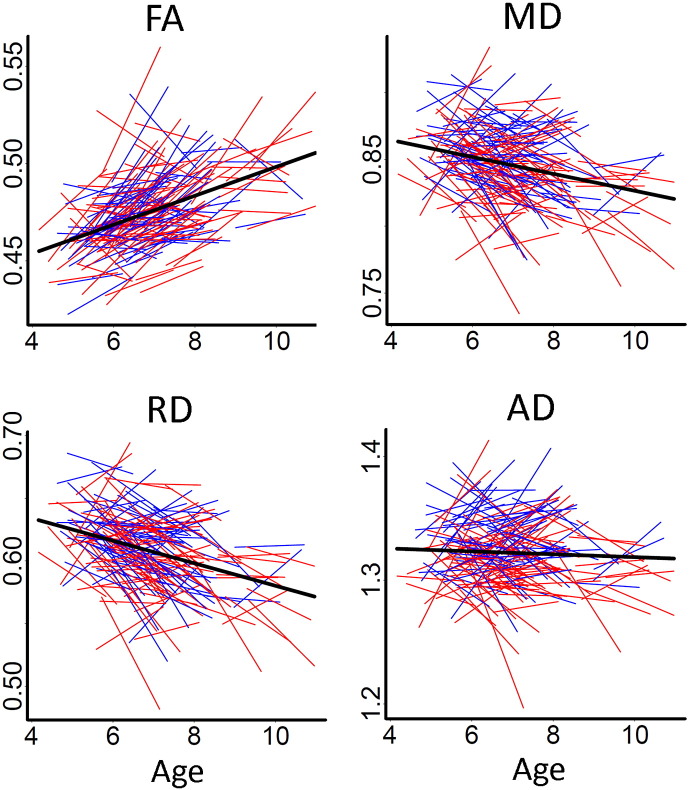
*Spaghetti plots of change in mean FA, MD, RD and AD with age.* Spaghetti plots of individual participant change in mean FA, MD, RD and AD with age (years). Females are plotted in red and males in blue. For each measure, an assumption-free general additive mix model as a function of age was fitted to accurately describe changes across the age range. Diffusivity values for MD, RD and AD are in 10^− 3^ x mm^2^/s.

Fig. 2 shows the relationship between mean FA/MD/RD/AD and age, when both cross-sectional and longitudinal information is taken into account by use of GAMM. The spaghetti plots indicated a linear increase for mean FA and linear decrease for mean MD, RD and AD. The decrease in mean AD was weak compared to RD and MD.

### WM tracts

**Fig. 3:**
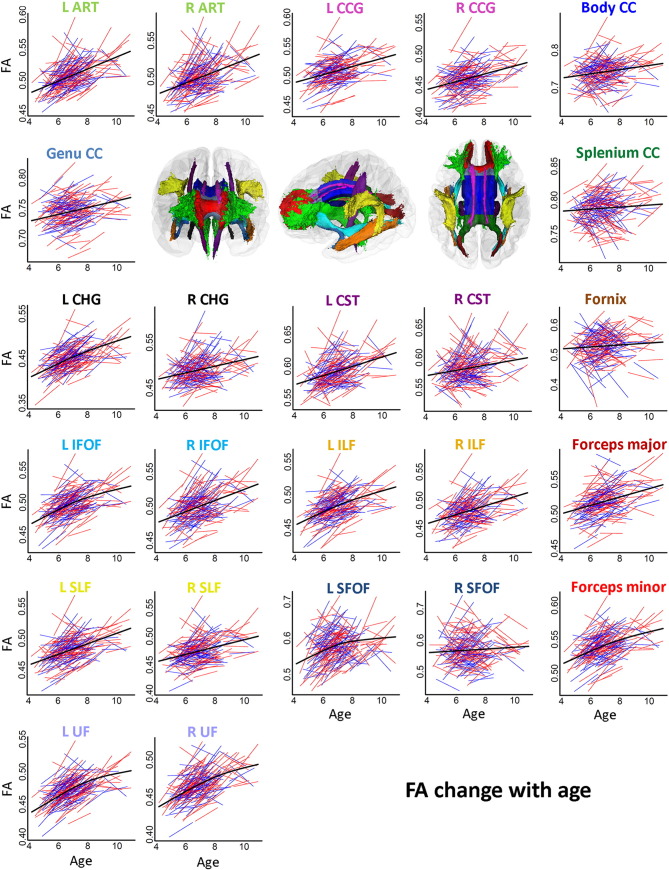
*Spaghetti plots of change for FA in specific tracts with age.* Spaghetti plots of individual participant change in FA in specific tracts with age (years). Females are plotted in red and males in blue. For each measure, an assumption-free general additive mixed model as a function of age was fitted to accurately describe changes across the age range. Three-dimensional renderings of the probabilistic tracts illustrate fifteen atlas-based probabilistic tracts from the Mori atlas in anterior, left, and dorsal views, displayed on a semitransparent template brain from FreeSurfer (fsaverage). The color-coded titles for each scatterplot represent the color of each specific WM tract. Color codes refer to: Light green: Anterior thalamic radiation (ATR), Pink: Cingulum-cingulate gyrus (CCG), Blue: Body of corpus callosum (CC Body), Gray-blue: Genu of corpus callosum (CC Genu), Green: Splenuim of corpus callosum (Splenium CC), Black: Cingulum-hippocampus gyrus (CHG), Purple: Cortico-spinal tract (CST), Brown: Fornix, Light blue: Inferior fronto-occipital fasciculus (IFOF), Orange: Inferior longitudinal fasciculus (ILF), Dark red: Forceps major, Yellow: Superior longitudinal fasciculus (SLF), Dark blue: Superior fronto-occipital fasciculus (SFOF), and Blue-purple: Uncinate fasciculus (UF). The 3D figures were made by the use of Slicer (http://www.slicer.org/).

**Fig. 4:**
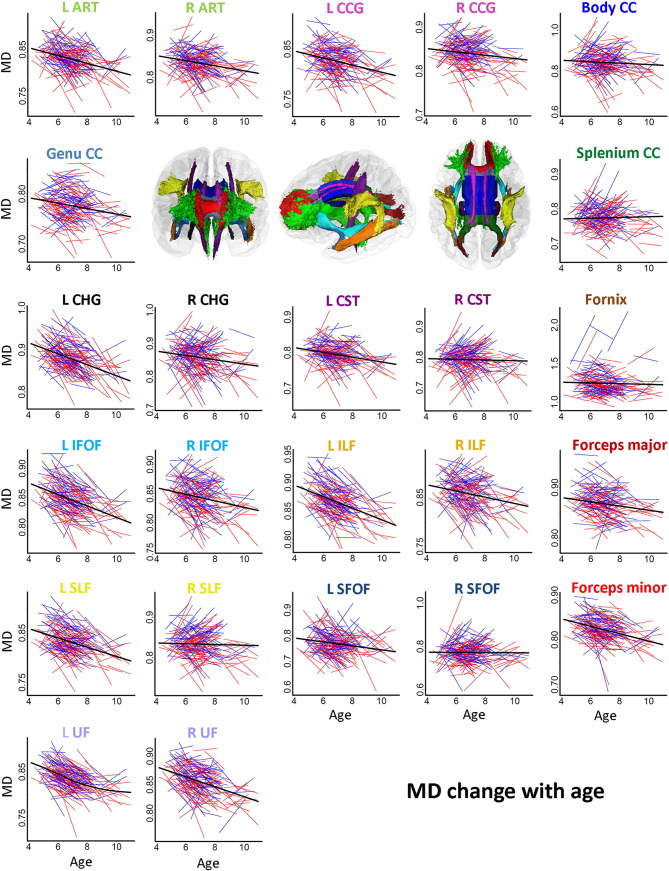
*Spaghetti plots of change for MD in specific tracts with age.* Spaghetti plots of individual participant change in MD in specific tracts with age (years). Females are plotted in red and males in blue. For each measure, an assumption-free general additive mixed model as a function of age was fitted to accurately describe changes across the age range. Diffusivity values for MD are in 10^− 3^ x mm^2^/s. Diffusivity values for MD are 10^− 3^ x mm^2^/s. Three-dimensional renderings of the probabilistic tracts illustrate fifteen atlas-based probabilistic tracts from the Mori atlas as for Fig. 3.

**Fig. 5:**
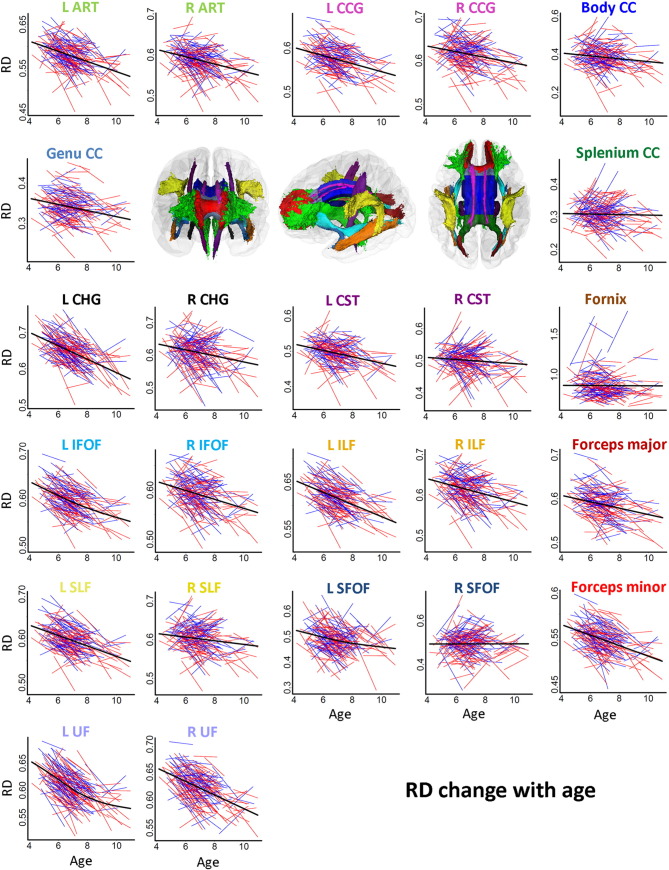
*Spaghetti plots of change for RD in specific tracts with age.* Spaghetti plots of individual participant change in RD in specific tracts with age (years). Females are plotted in red and males in blue. For each measure, an assumption-free general additive mixed model as a function of age was fitted to accurately describe changes across the age range. Diffusivity values RD are in 10^-3^ x mm^2^/s. Diffusivity values RD are 10^− 3^ x mm^2^/s. Three-dimensional renderings of the probabilistic tracts illustrate fifteen atlas-based probabilistic tracts from the Mori as for Fig. 3.

**Fig. 6:**
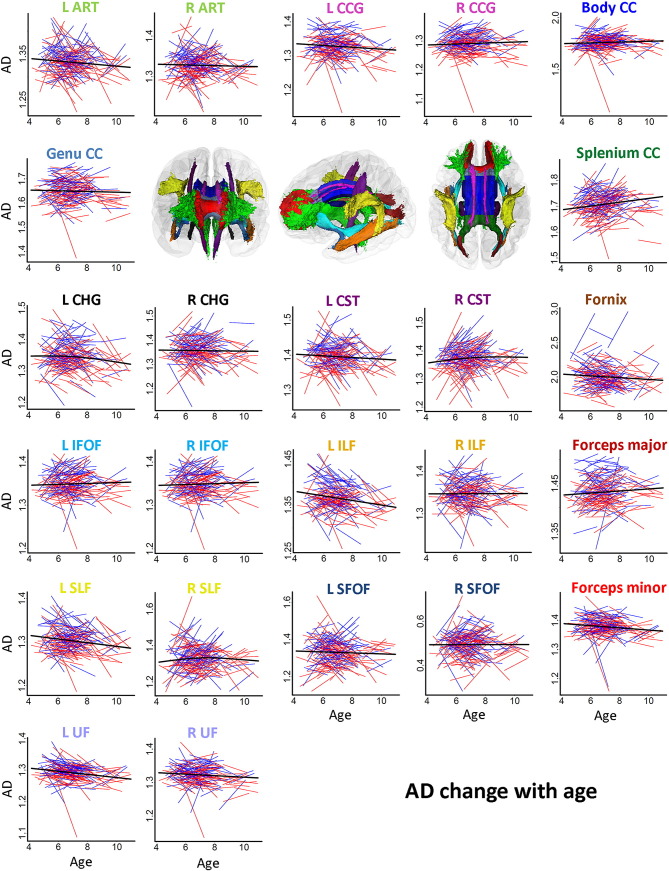
*Spaghetti plots of change for AD in specific tracts with age.* Spaghetti plots of individual participant change in AD in specific tracts with age (years). Females are plotted in red and males in blue. For each measure, an assumption-free general additive model as a function of age was fitted to accurately describe changes across the age range. Diffusivity values for AD are in 10^− 3^ x mm^2^/s. Diffusivity values for AD are 10^− 3^ x mm^2^/s. Three-dimensional renderings of the probabilistic tracts illustrate fifteen atlas-based probabilistic tracts from the Mori atlas as for Fig. 3.

Spaghetti plots of individual participant change from tp1 to tp2 in FA, MD, RD and AD in WM tracts are displayed in Fig. 3, Fig. 4, Fig. 5, Fig. 6, respectively. Ten out of fifteen WM tracts showed linear development for FA, fourteen for MD, and twelve for RD and AD. Specifically, non-linear trajectories were found for FA in forceps minor, left IFOF, left ILF, left SFOF, and left and right UF, for MD in left UF, for RD in left IFOF, left SFOF, left UF and for AD in left CHG, right CST and right SLF. These tracts all showed a deceleration of change with age.

APC (Table 4) was positive for FA in all WM tracts (ranging from 0.05 to 3.85), negative in thirteen out of fifteen tracts for MD (ranging from − 0.13 to − 1.88) and RD (ranging from − 0.24 to − 3.8). For AD, APC was negative in nine tracts in the left hemisphere (ranging from − 0.17 to − 0.60) and positive in seven tracts in the right hemisphere (ranging from 0.10 to 1.09). The APC for WM tracts also showed regional differences between linear developmental patterns; FA in CC splenium (APC = 0.05) and AD in CC genu (APC = − 0.03) showed the lowest APC among all WM tracts. Small positive APC (< 1.0%) was found for FA in right CST, right SFOF, CC body, CC genu and fornix, for MD in right CST, right SLF, right SFOF, CC splenium and fornix, and for RD in right CST and CC splenium. Small negative APC (>− 1.0%) was found for MD in right ATR, left and right CCG, right CHG, left CST, right IFOF, right ILF, CC body, CC genu and forceps major, and for RD in right CCG and right SLF. For AD, APC > 1.0%/≥ 1.0% was only found in right SFOF (APC = 1.09). Larger changes with APC > 2% were found for FA (ranging from 2.13 to 3.85) in left ATR, left CHG, left IFOF, left ILF, left SFOF and left and right UF, and APC ≥ 2.0% for RD (ranging from − 2.00 to − 3.82) in left ATR, left CHG, left IFOF, left ILF, left SLF, left SFOF, left and right UF, CC genu and forceps minor. No APC ≥ 2.0% was found for MD or AD in tracts. Results from the paired t-tests comparing differences between APC in tracts and global APC showed significant regional differences in change rates (see Online Supplementary Table 1).

### WM development across gradients

**Fig. 7:**
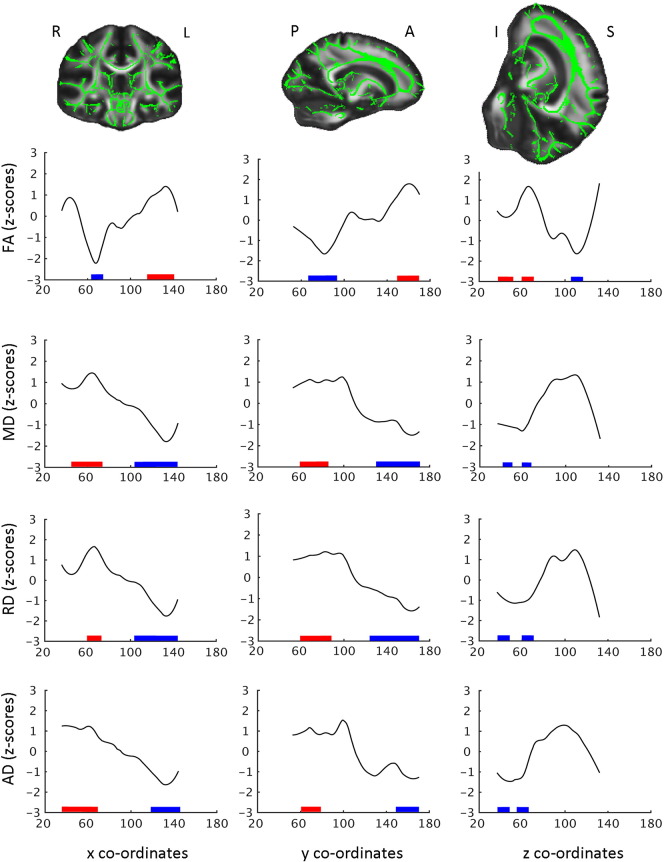
*Slice-by-slice profiles of mean change for FA, MD, RD and AD.* Slice-by-slice profiles of mean change for FA, MD, RD and AD, plotted for each coronal, sagittal and axial slice. X, Y and Z co-ordinates in MNI Space. Change for FA, MD, RD and AD are z-transformed change values. The FA skeleton (green) displayed on the FMRIB FA template in MNI space illustrate the principal gradients. L = left hemisphere, R = right hemisphere, P = posterior, A = anterior, I = inferior and S = superior. Significant (p < 0.05) differences between mean change and change for each slice are marked at the x axis. Red signifies greater positive change relative to mean change and blue signifies greater negative change relative to mean change. Slices marked are > 5 continues significant (p < 0.05) slices showing difference in change relative to mean change.

Z-transformed change values for each metric across x, y and z co-ordinates are plotted in Fig. 7. The posterior-to-anterior gradients displayed a lesser-to-greater change for FA, MD, RD and AD, and greater change in anterior compared to posterior regions was seen for all metrics (FA: t = − 5.82/MD: t = 8.33/RD: t = 8.23/AD: t = 7.48, all p's < 0.001). The inferior-to-superior gradient displayed a U-shaped pattern for FA, and inverted U-shaped patterns for MD, RD and AD, illustrating greater change in specific superior and inferior regions. Significant (p < 0.001) differences were found between change in inferior and superior ROIs for all DTI metrics (FA: t = 4.97/MD: t = − 5.21/RD: t = − 5.00/AD: t = − 4.98), where all showed greater change in the inferior region compared to the superior region. No consistent medial-to lateral pattern was observed for any of the metrics. Further statistical testing was therefore not performed.

### Influence of sex and hemisphere

The GLMs showed no significant (p < 0.05) effect of sex on change for global FA, MD, RD, AD or in specific WM tracts (ranging from p = .101 to p < .999), when controlling for age, motion at both time points and interval. For RD in forceps minor there was a trend toward and interaction of change and sex (F = 3.30, p ≤ .084) with more change for female compared to males. To test for hemisphere differences in changes in FA, MD, RD and AD for bilateral tracts, we performed paired-samples t-tests (Table 5). Significantly corrected (p < 0.006) greater change for left hemisphere was seen for almost all WM tracts. FA in ILF was significant at p < 0.05 (t = 2.26, p = .025), but would not survive a strict correction for number of comparison. WM tracts not significant at p < 0.05 were: FA in CCG (t = 1.68, p = .095), and AD in CCG (t = − 1.14, p = .257), showing no significant difference in change between hemispheres. Also, table 4 shows APC for left and right hemisphere for all bilateral WM tracts and indicates hemisphere differences with an overall greater change in left hemisphere.

## Discussion

We found significant increase in global FA and decreases in global MD and RD over time in 4–11 year olds, and these global changes did not vary as a function of age. For global AD, the development of WM was more complex. Importantly, eight specific WM tracts showed non-linear developmental patterns for one or several diffusion metrics. There were differences in change rates between WM tracts. In addition, the results suggested that WM development follows major gradients in the brain in addition to these regional changes in WM tracts.

Our main results are consistent with the few available previous longitudinal developmental studies also finding FA to increase and MD and RD to decrease through childhood and adolescence, while reporting less consistent findings for AD (Bava et al., 2010, Lebel and Beaulieu, 2011, Brouwer et al., 2012, Simmonds et al., 2014). As noted by Concha (2014), the interpretation rests on knowledge of what is known to drive diffusion anisotropy, namely axonal membranes, density and coherence, as well as myelin sheaths. FA and MD reflect a variety of microstructural features, including the relative alignment of individual axons, their diameter and thickness of the myelin sheath, as well as axonal density (Beaulieu, 2002). Animal studies indicate that RD is related to myelination and axonal packing (Beaulieu, 2002, Song et al., 2002), and RD has been found to positively correlate with the mean axon diameter while correlating negatively with AD (Barazany et al., 2009). Although biophysical processes associated with normal human development are more complex than in these animal models (Schmierer et al., 2007, Concha et al., 2010), the myelination process in children may lead to decrease in RD along with an increase in FA (Bonekamp et al., 2007, Eluvathingal et al., 2007, Lebel et al., 2008, Faria et al., 2010, Rose et al., 2014, Uda et al., 2015). Neural activity together with experiences during development may influence myelination and possibly contribute to the observed diffusion parameter changes (Demerens et al., 1996, Ishibashi et al., 2006, Sampaio-Baptista et al., 2013).

### Age-related change

Results showed no effect of age on global change within the current age span. Non-linear relationships between different DTI metrics and age have been found in longitudinal data (Lebel and Beaulieu, 2011), and cross-sectional studies have shown nonlinear grown patterns for several WM tracts in participants 0 to 11 years of age (Mukherjee et al., 2001) and 5 to 30 years of age (Lebel et al., 2008). The present study was focused on pre-adolescence childhood, with very dense sampling from 4 to 11 years. Most likely, the global changes in WM microstructure in this age period are rather stable. With the relatively short age-range, this did not allow reliable detections of deviations from linearity. For instance, inspections of the curves from Lebel and Beaulieu (2011) show that even though highly non-linear relationships are found for the wider age-range of 5 to 32 years, the curves for the pre-adolescence period seem mainly linear, in accordance with the present findings. Also, the steep nonlinear increase with age from Mukherjee et al. (2001) was mostly observed before the age of four. Thus, the present study indicates that 4–11 years is a period of rapid development of WM, with the gradual reduction in rate of change expected later in adolescence not yet being observed in global measures.

Despite the linear changes in the global measures, non-linear trajectories with a deceleration of change with age were observed for a number of specific WM tracts. This was found for FA in forceps minor, left CHG, left IFOF, left ILF, left SFOF, and left and right UF, MD in left UF, RD in left IFOF, left SFOF and left UF, and AD in left CHG, right CST and right SLF. Here, greater APC was observed relative to global APC for FA, MD and RD. The small APC found in CC splenium for all DTI metrics are supported by previous autopsy studies showing early maturation in this specific WM tract before the age of four. Association tracts have been suggested to develop later and are in accordance with the greater APC found in CHG, ILF, IFOF, SLF, SFOF and UF relative to global APC for all DTI metrics in the current study (Brody et al., 1987, Kinney et al., 1988). Some developmental studies have investigated the relationships between WM microstructure and cognitive function longitudinally (see e.g (Yeatman et al., 2012, Treit et al., 2013, Gautam et al., 2014, Ullman et al., 2014)), and to some extent, there is evidence that observed regional difference in developmental patterns for WM tracts might be associated with development of higher level cognitive functions (Nagy et al., 2004, Walsh et al., 2011, Østby et al., 2011, Yeatman et al., 2012, Klarborg et al., 2013, Treit et al., 2013, Gautam et al., 2014, Peters et al., 2014, Ullman et al., 2014).

### WM development across gradients

Within the age range from 4 to 11 years, we found more change in anterior than posterior regions for all metrics. This suggests that development of WM microstructure may follow major gradients in the brain in addition to individual WM tracts. The developmental pattern observed across posterior-to-anterior gradients is in agreement with a cross sectional DTI finding (Colby et al., 2011), a developmental study examining WM development (Tzarouchi et al., 2009), and Westlye et al. (2010a) showing that intra-cortical T1 signal intensity follows a posterior-to-anterior gradient from childhood throughout life. The larger anterior change may indirectly be related to early and primary neurobiological mechanisms such as synapse elimination and maturational myelination at different stages in development (Huttenlocher, 1990, Huttenlocher and Dabholkar, 1997). White matter development, including myelination has been found to start prenatally, during infancy it develops from posterior-to-anterior, inferior-to-superior, and central-to-peripheral regions (Barkovich et al., 1988, Bendersky et al., 2006, de Graaf-Peters and Hadders-Algra, 2006), and it continues into adulthood (Paus et al., 1999, Bartzokis et al., 2001, Sowell et al., 2002). Basic neurobiological mechanisms such as myelination and axonal development may have brain correlates that can be detected by DTI, and as such these principles could partly impact the maturational changes in WM DTI metrics observed in the present study. In an adult longitudinal DTI study, posterior–anterior gradients were found to increase gradually but the gradients were anatomically specific rather than global, and age-related changes appeared to be principally governed by inferior-to-superior gradients (Sexton et al., 2014). The current results does not suggest that WM development from 4 to 11 years proceeds in a continuous fashion from inferior to superior regions, but indicate greater anterior changes. While we have mentioned some possible neurobiological mechanisms that might underlie changes in DTI metrics (Barkovich et al., 1988, Song et al., 2002, Bendersky et al., 2006, de Graaf-Peters and Hadders-Algra, 2006, Barazany et al., 2009), we do not believe that sufficient evidence exists to enable direct interpretation of the causes of changes along these gradients specifically.

### Influence of sex and hemisphere

The results showed no significant sex differences in change for global FA, MD, RD, AD or in specific WM tracts. DTI studies show mixed findings on the interaction between age and sex, and this is an area for future research (Bava et al., 2010, Giorgio et al., 2010, Lebel and Beaulieu, 2011, Wang et al., 2012, Simmonds et al., 2014). The lateralization analyses showed a greater change for FA, MD, RD and AD in all WM tracts in left hemisphere. Hemisphere effects were also illustrated by APC for bilateral tracts indicating hemisphere differences with an overall larger change in the left hemisphere for most tracts. In the literature, reports on hemispheric specificity of DTI parameters have been inconsistent (Park et al., 2004). Lateral asymmetry in FA has been reported with greater FA values of the left hemisphere (Eluvathingal et al., 2007) but also greater FA values of the right hemisphere (Uda et al., 2015).

### Limitations and future directions

Participants generally performed above average on tests of cognitive functioning, and may not be representative of the general population. With regard to data acquisition and analysis, the study benefitted from the same scanner and sequence being used at both time points. Although drift in scanner performance over time is possible, and measures could be affected by MRI software upgrades, it is unlikely that such factors could explain the pattern of our findings. Because DTI measures are highly sensitive to motion artifacts, only participants deemed to have no or minimal movement artifacts at both time points were included in the analyses. Excluding participants not able to complete the scan or not being able to lie still in the scanner may have impact upon our results. In the current study, the threshold for the mean FA skeleton was set at 0.25 and the nonlinear alignment was deemed successful. Even so, there are limitations regarding partial voluming in the borders between WM and subcortical areas such as thalamus (Smith et al., 2006). Further, care must be taken as the alignment between the study data and the template/atlas space may be inaccurate, possible leading to incorrect conclusions about location (Johansen-Berg and Behrens, 2013). All WM tracts were therefore manually checked and deemed satisfactory at probability threshold of 5%.

In regions with crossing fibers, FA may be influenced by the number and direction of these fiber tracts, complicating the biological interpretations. Even though some methods do estimate and differentiate multiple fibers in each voxel (Tuch et al., 2003, Wedeen et al., 2005, Behrens et al., 2007), this was not done for the current study. It must also be noted that the results from the spatial gradient are not independent of the developmental patterns for specific WM tracts. For instance, association fibers tend to occupy most lateral regions, while callosal projections occupy medial regions. This must be taken into account when interpreting the gradient results. Gradients were measured from the FA skeleton, which means that only a fraction of the WM is included in these analyses. Further, limitations regarding partial voluming, including some gray matter in the skeleton might also influence the results of gradients. Alternative approaches including a larger part of WM could conceivably have given partly different results. The gradients were raw average change scores, and we did not test for potential effects of covariates of no interest. Although we believe these effects to be relatively small, based on the others analyses performed, interpretations should be made with this limitation in mind. Further longitudinal investigations should also explore WM microstructure development in relation to change in cognitive abilities and between-subject variability in pre- and postnatal environmental factors and genotypes.

## Conclusion

In conclusion, we have shown extensive longitudinal changes in WM microstructure in a large pre-adolescence sample. Interestingly, for the most part, the global changes observed seem to be of equivalent magnitude at different ages from 4 to 11 years, indicating that this is an age period of rapid and rather constant WM change. It should be noted, though, that non-linear trajectories with a deceleration of change with age were observed for a number of specific WM tracts. The most widespread developmental changes in the WM skeleton were found for FA and RD, with even greater change in the anterior compared to the posterior region, and in the inferior compared to the superior region. The present study hence shows both age-invariant global patterns and considerable regional differences in white matter change in the age range 4–11 years.

The following are the supplementary data related to this article.

**Online Supplementary Fig. 1:**
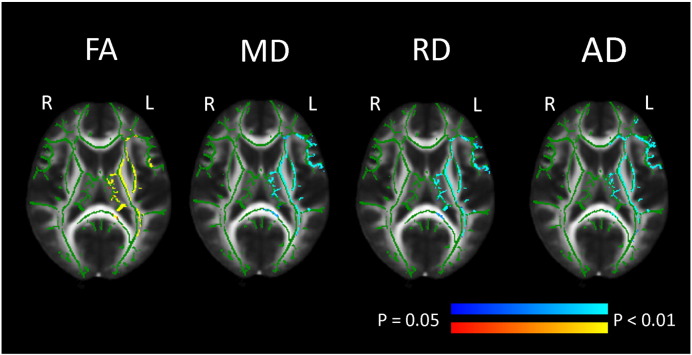
Title: Global hemisphere differences for FA, MD, RD and AD. Global hemisphere differences showing significantly (p < 0.05, correction for multiple comparisons across space) greater positive change (yellow and red) in left hemisphere compared to right hemisphere (L > R) for FA, and greater positive change (blue and light blue) in the right hemisphere compared to left hemisphere (L < R) in MD, RD and AD. Results are overlaid on the FA symmetrized skeleton (green), displayed on the FMRIB FA template in MNI space. L = left hemisphere and R = right hemisphere. Z MNI coordinates = 85.

Online supplementary Table 1 Comparing annual percentage change between WM tracts and global skeleton for FA, MD, RD and AD.

## Conflict of interest

The authors declare no competing financial interests.

## Figures and Tables

**Table 1:** Participant characteristics and demographics.

	Mean	Median	SD	Range
N (females: males)	159 (90: 69)			
Age tp1	6.2	6.0	1.1	4.2–9.3
Age tp2	7.8	7.6	1.1	5.8–11.0
Interval days	584	589	52	456–819
Mean scaled score WPPSI tp1^a^	11.6	11.9	1.9	6–16
Mean scaled score WPPSI tp2^b^	12.3	12.4	1.3	11–15
Estimated IQ WASI tp1^c^	109.9	109.0	11.7	77–140
Estimated IQ WASI tp2^d^	108.8	107.0	12.1	77–141

Participants ≤ 6.5 years of age completed the vocabulary, similarities, block-design and matrix subtests of the Wechsler Preschool and Primary Scale of Intelligence (WPPSI–III) (Wechsler, 2002). Participants > 6.5 years of age were tested using the vocabulary, similarities, block-design and matrix subtests of the Wechsler Abbreviated Scale of Intelligence (WASI) (Wechsler, 1999). Mean scaled score WPPSI = Mean of available scaled scores form 4 WPPSI subtests. Number of participants; ^a^n = 102, ^b^n = 14 ^c^n = 54, ^d^n = 141.

**Table 2:** Number and percentage of significant voxels.

	Positive change		Negative change	
	No. of sig. voxels	% sig. voxels	No. of sig. voxels	% sig. voxels
FA	111,907	73	206	0.1
MD	7425	5	77,708	51
RD	2559	2	102,136	67
AD	25,818	17	26,934	18

The table shows number and percentage of significant voxels (p < 0.05, correction for multiple comparisons across space) in the FA skeleton for both positive and negative change with age, sex, motion at both time point and interval as confound regressors. The FA skeleton consisted of 152,284 WM voxels.

**Table 3:** Relationship between FA, MD, RD and AD and age.

	Time point 1		Time point 2		Change (tp2 − tp1)	
	r	p	r	p	r	p
FA	**.34**	**< .001**	**.30**	**< .001**	− .05	.516
MD	**− .27**	**< .001**	*− .24*	*.003*	.03	.756
RD	**− .31**	**< .001**	**− .27**	**< .001**	.03	.736
AD	− .13	.132	− .13	.104	.02	.814

Table shows partial correlation (r) between FA, MD, RD and AD at tp1, tp2 and change (time point 2 − time point 1) and age, controlling for motion at each time point and sex. Additionally, motion at both time points and interval were controlled for when testing change. Significant changes at p < 0.05 are shown in italic and significant changes at p < 0.001 are shown in bold.

**Table 4:** Annual percentage change for FA, MD, RD and AD.

	FA		MD		RD		AD	
	Mean %		Mean %		Mean %		Mean %	
	L	R	L	R	L	R	L	R
Global	1.80		− 0.73		− 1.49		− 0.02	
ATR	2.24*	1.94*	− 1.28*	− 0.66	− 2.33*	**− 1.52**	− 0.34*	0.13*
CCG	1.50*	1.43*	**− 0.82**	− 0.23*	**− 1.54**	− 0.79*	**− 0.17**	0.32*
CHG	3.84*	1.75	− 1.80*	**− 0.76**	− 3.29*	− 1.47	− 0.30*	**− 0.07**
CST	1.38*	0.61*	**− 0.89**	0.42*	**− 1.76**	0.24*	− 0.24*	0.60*
IFOF	2.15*	1.76	− 1.30*	− 0.59	− 2.32*	− 1.46	− 0.37*	0.20*
ILF	2.13*	**1.81**	− 1.29*	− 0.69	− 2.25*	**− 1.54**	− 0.40*	0.10
SLF	1.96*	1.42	− 1.27*	0.21*	− 2.15*	− 0.43*	− 0.44*	0.83*
SFOF	3.85*	0.98*	− 1.88*	0.98*	− 3.82*	1.38*	**− 0.23**	1.09*
UF	2.79*	3.32*	− 1.74*	− 1.02*	− 2.92*	− 2.00*	− 0.60*	**− 0.06**
CC Body	0.88*		− 0.13*		− 1.33		0.55*	
CC Genu	0.91*		**− 0.78**		− 2.44*		**− 0.03**	
CC Splenium	0.05*		0.77*		0.89*		0.78*	
Forceps major	1.13*		− 0.35*		− 1.03*		0.22*	
Forceps minor	1.76		− 1.14*		− 2.24*		− 0.25*	
Fornix	0.53*		0.21		1.12*		**− 0.39**	

Annual percentage change for global FA, MD, RD and AD and all WM tracts. L = left hemisphere and R = right hemisphere. ATR = Anterior thalamic radiation, CCG = Cingulum-cingulate gyrus, CHG = Cingulum-hippocampus gyrus, IFOF = Inferior fronto-occipital fasciculus, ILF = Inferior longitudinal fasciculus, SLF = Superior longitudinal fasciculus, SFOF = Superior fronto-occipital fasciculus, UF = Uncinate fasciculus and CC = corpus callosum. Numbers in bold signify greater APC relative to global APC. * signify that APC for the tract is significantly (p < 0.05) different from global APC (see Supplementary Table 1).

Annual percentage change for global FA, MD, RD and AD and all WM tracts. L = left hemisphere and R = right hemisphere. ATR = Anterior thalamic radiation, CCG = Cingulum-cingulate gyrus, CHG = Cingulum-hippocampus gyrus, IFOF = Inferior fronto-occipital fasciculus, ILF = Inferior longitudinal fasciculus, SLF = Superior longitudinal fasciculus, SFOF = Superior fronto-occipital fasciculus, UF = Uncinate fasciculus and CC = corpus callosum. Numbers in bold signify greater APC relative to global APC. * signify that APC for the tract is significantly (p < 0.05) different from global APC (see Supplementary Table 1).

**Table 5:** Hemisphere differences for all WM tracts.

	Hemisphere difference (L-H)							
	FA		MD		RD		AD	
	t	p	t	p	t	p	t	P
ATR	**3.93**	**< .001**	**− 6.79**	**< .001**	**− 6.33**	**< .001**	**− 6.65**	**< .001**
CCG	1.68	.095	**− 5.02**	**< .001**	**− 4.25**	**< .001**	− 1.14	.257
CHG	**6.75**	**< .001**	**− 3.99**	**< .001**	**− 5.37**	**< .001**	**− 1.14**	**< .001**
CST	**5.29**	**< .001**	**− 6.27**	**< .001**	**− 6.26**	**< .001**	**− 5.89**	**< .001**
IFOF	**3.35**	**< .001**	**− 5.74**	**< .001**	**− 5.10**	**< .001**	**− 6.34**	**< .001**
ILF	*2.26*	*.025*	**− 3.89**	**< .001**	**− 3.37**	**< .001**	**− 4.55**	**< .001**
SLF	**4.73**	**< .001**	**− 8.05**	**< .001**	**− 7.55**	**< .001**	**− 8.42**	**< .001**
SFOF	**6.16**	**< .001**	**− 7.08**	**< .001**	**− 7.20**	**< .001**	**− 4.13**	**< .001**
UF	**4.35**	**< .001**	**− 6.49**	**< .001**	**− 6.12**	**< .001**	**− 5.75**	**< .001**

The significance of hemisphere differences in change in FA, MR, RD and AD in all bilateral tracts were tested with paired-samples t-tests. L = left hemisphere; R = right hemisphere. ATR = Anterior thalamic radiation, CCG = Cingulum-cingulate gyrus, CHG = Cingulum-hippocampus gyrus, IFOF = Inferior fronto-occipital fasciculus, ILF = Inferior longitudinal fasciculus, SLF = Superior longitudinal fasciculus, SFOF = Superior fronto-occipital fasciculus and UF = Uncinate fasciculus. Significant changes at p < 0.05 are shown in italic and significant changes at p < 0.001 are shown in bold.
