# Supplementary material for: Changes in white matter microstructure in the developing brain—A longitudinal diffusion tensor imaging study of children from 4 to 11 years of age
Source: Neuroimage. 2016 Jan 1;124(Pt A):473–86. doi: 10.1016/j.neuroimage.2015.09.017 (PMC4655940; doi:10.1016/j.neuroimage.2015.09.017)
Supplement: Online supplementary Table 1 — Comparing annual percentage change between WM tracts and global skeleton for FA, MD, RD and AD. [file mmc1.docx]

**Online supplementary Table 1. Comparing annual percentage change between WM tracts and global skeleton for FA, MD, RD and AD**

| APC in tracts - Global APC | | | | | | | | | |
| --- | --- | --- | --- | --- | --- | --- | --- | --- | --- |
|  |  | FA | | MD | | RD | | AD | |
| Tracts | Hemi | t | p | t | p | t | p | t | P |
| ATR | left | **6.35** | **<.001** | **-6.16** | **<.001** | **-7.17** | **<.001** | **-4.61** | **<.001** |
|  | right | *2.07* | *.040* | .89 | .375 | **-**.29 | .773 | *2.21* | *.029* |
| CCG | left | **-3.29** | **<.001** | **-**.92 | .360 | **-**.40 | .688 | **-**1.81 | .073 |
|  | right | **-3.64** | **<.001** | **5.25** | **<.001** | **5.58** | **<.001** | **4.28** | **<.001** |
| CHG | left | **11.54** | **<.001** | **-6.87** | **<.001** | **-9.00** | **<.001** | **-***2.03* | *.044* |
|  | right | **-**.22 | .825 | **-**.11 | .913 | .07 | .943 | **-**.35 | .730 |
| CST | left | **-4.77** | **<.001** | **-**1.34 | .184 | **-**1.35 | .179 | **-***2.63* | *.009* |
|  | right | **-10.25** | **<.001** | **5.49** | **<.001** | **5.33** | **<.001** | **4.09** | **<.001** |
| IFOF | left | **3.59** | **<.001** | **-4.76** | **<.001** | **-6.00** | **<.001** | **-3.64** | **<.001** |
|  | right | **-**.487 | .627 | 1.73 | .085 | .24 | .808 | **3.39** | **<.001** |
| ILF | left | *3.09* | *.002* | **-4.36** | **<.001** | **-4.80** | **<.001** | **-3.51** | **<.001** |
|  | right | .14 | .890 | .39 | .699 | **-**.313 | .755 | 1.42 | .158 |
| SLF | left | *2.10* | *.037* | **-5.19** | **<.001** | **-***5.07* | *.037* | **-5.13** | **<.001** |
|  | right | **-4.76** | **<.001** | **8.64** | **<.001** | **7.96** | **<.001** | **9.20** | **<.001** |
| SFOF  UF | left | **5.22** | **<.001** | **-3.34** | **<.001** | **-4.08** | **<.001** | **-**.80 | .424 |
|  | right | **-***2.38* | *.019* | **4.92** | **<.001** | **4.48** | **<.001** | **3.83** | **<.001** |
|  | left | **7.94** | **<.001** | **-7.03** | **<.001** | **-7.64** | **<.001** | **-5.39** | **<.001** |
|  | right | **4.58** | **<.001** | **-***2.31* | *.022* | **-***3.19* | *.002* | -.41 | .681 |
| CC Body | | **-6.61** | **<.001** | *2.06* | *.042* | .21 | .838 | *2.83* | *.005* |
| CC Genu | | **-6.44** | **<.001** | **-**.33 | .742 | **-3.52** | **<.001** | **-**.063 | .950 |
| CC Splenium | | **-13.09** | **<.001** | **8.00** | **<.001** | **4.04** | **<.001** | **6.67** | **<.001** |
| Forceps major | | **-5.70** | **<.001** | *2.96* | *.004* | *2.81* | *.006* | *2.29* | *.023* |
| Forceps minor | | **-**.31 | .756 | **-***2.99* | *.003* | **-4.42** | **<.001** | **-***2.03* | *.044* |
| Fornix | | **-***3.00* | *.004* | 1.88 | .063 | **3.63** | **<.001** | **-**.87 | 383 |

Table shows paired t-test for all DTI metrics between APC in each tract and global APC. APC = Annual percentage change. ATR = Anterior thalamic radiation, CCG = Cingulum-cingulate gyrus, CHG = Cingulum-hippocampus gyrus, IFOF = Inferior fronto-occipital fasciculus, ILF = Inferior longitudinal fasciculus, SLF = Superior longitudinal fasciculus, SFOF = Superior fronto-occipital fasciculus, UF = Uncinate fasciculus and CC = corpus callosum. Significant changes at p < 0.05 are shown in italic and significant changes at p < 0.001 are shown in bold. For FA the negative t-statistic means less APC in tracts relative to global APC, and for MD, RD and AD the negative t-statistic means greater decreased APC in tracts relative to global APC.
